# Variants in *RHOBTB2* associated with cancer and rare developmental and epileptic encephalopathy

**DOI:** 10.3389/fped.2024.1448793

**Published:** 2024-12-16

**Authors:** Elaina Solano, Aleksandra Foksinska, Camerron M. Crowder

**Affiliations:** ^1^ Department of Neurobiology, University of Alabama at Birmingham, Birmingham, AL, United States; ^2^ Hugh Kaul Precision Medicine Institute, University of Alabama at Birmingham, Birmingham, AL, United States

**Keywords:** neurodevelopmental disorder, developmental and epileptic encephalopathy, *RHOBTB2*-related disorders, cancer, tumor suppressor, precision medicine, genetic variants

## Abstract

RHOBTB2 is a member of the Rho GTPases subfamily of signaling proteins, known tumor suppressors whose loss of function and decreased expression is associated with cancer onset. Beyond its cancer-related role, RHOBTB2 is implicated in rare neurodevelopmental disorders, specifically *RHOBTB2*-related disorders, recognized in 2018 as a subtype of developmental and epileptic encephalopathies (DEE). Common symptoms of these disorders include early-onset epilepsy, severe intellectual disability, microcephaly, and movement disorders. Few studies have investigated patient variants associated with *RHOBTB2*-related disorders, and the impact of these variants on protein function remains unclear. Limited research suggests that the accumulation of RHOBTB2 in neural tissues contributes to the development of DEE. Similarly, preclinical studies indicate that missense variants near or in the BTB domain of RHOBTB2 result in decreased degradation of RHOBTB2 and the onset of DEE, whereas variants in the GTPase domain cause more variable neurodevelopmental symptoms, but do not impair proteasomal degradation of RHOBTB2. However, the exact pathophysiological mechanisms are unclear and may differ across variants. Current treatment approaches for individuals with *RHOBTB2*-related DEE involve the use of antiseizure medications to decrease seizures; however, no treatments have been identified that address the other symptoms or the underlying pathophysiological mechanisms associated with these disorders. Overall, RHOBTB2 remains an understudied protein with limited information on its function and how it contributes to disease mechanisms. This review provides an overview of the current knowledge of RHOBTB2 function*,* with an emphasis on its association with neurodevelopmental disorders through an analysis of preclinical studies and case reports that link individual variants with clinical features.

## 1 Introduction

Neurodevelopmental disorders (NDDs) are heterogeneous conditions that arise from impairments in the growth, development, and function of the central nervous system (1). These disorders manifest early during a child's development and can affect various domains including cognition, behavior, motor function, and social interactions. An increasing number of *de novo* variants occurring in oncogenes and tumor suppressor genes are associated with rare disorders, and in particular, rare neurodevelopmental disorders (NDDs). The reverse also holds true, as individuals with rare NDDs may be at higher risk of developing certain cancers (2). For instance, *de novo* variants in DDX3X are associated with severe NDD, structural brain abnormalities, and intellectual disability, while somatic DDX3X variants are associated with aggressive cancers (3). Similarly, germline variants in FBXW7, a tumor suppressor, cause impaired ubiquitination and NDD (1). This overlap between NDD and cancer occurrence could be partly due to genes that are involved in signaling pathways shared between the two pathologies, such as MAPK and mTOR, which are crucial for cell proliferation and differentiation (4).

*RHOBTB2*, first identified as a tumor suppressor gene, has recently been implicated in a neurodevelopmental disorder. While there are some hypotheses surrounding how the RHOBTB2 protein is involved in cancer and NDD related to variant-specific consequences, the mechanism by which it contributes to these pathologies remains unknown. This review will cover an overview of the current understanding of the structure and function of RHOBTB2, followed by an analysis of recent studies highlighting its hypothesized role in cancer and NDD, clinical characteristics, treatment approaches, and current gaps in knowledge. Lastly, future research directions will be discussed, emphasizing the importance of further *in vivo* studies and the potential for exploring new treatment avenues targeting *RHOBTB2*-related pathologies. Focusing on understudied genes such as *RHOBTB2* exemplifies how deeper insights into monogenic disorders could provide answers for individuals affected by rare neurodevelopmental disorders and for those exploring novel precision oncology treatments.

## 2 Structure and function of *RHOBTB2*

**Figure 1:**
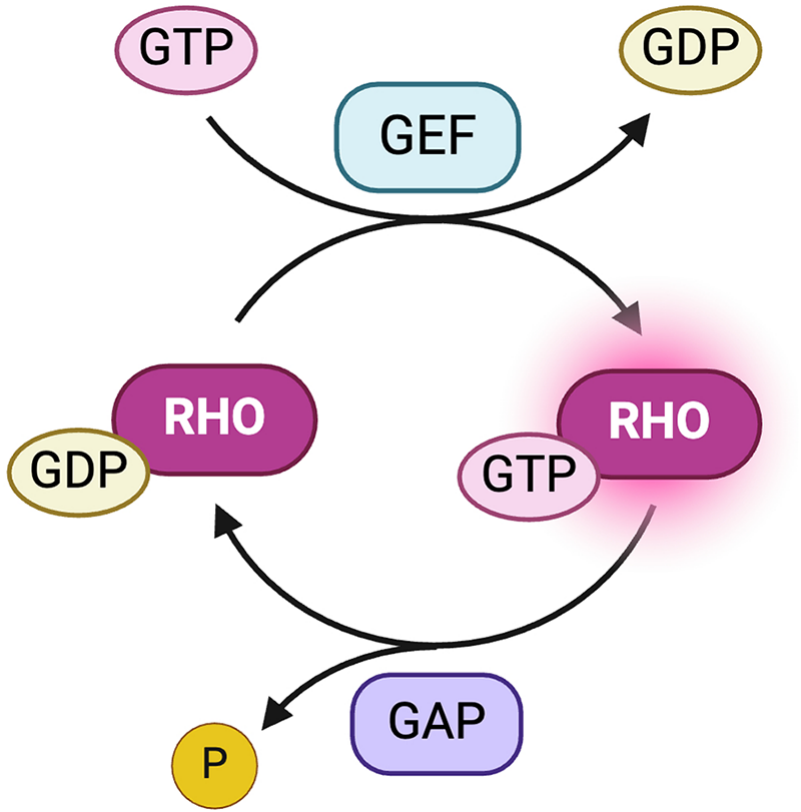
Activation and deactivation of Rho proteins. Guanine exchange factors (GEFs) catalyze the release of bound GDP, resulting in an active, GTP-bound Rho protein (7). GTPase-activating proteins (GAPs) hydrolyze GTP, releasing an inorganic phosphate (P_i_), to convert Rho proteins into the inactive form (7). Created with BioRender.com.

Rho-related BTB domain-containing protein 2 (RHOBTB2), encoded by the gene *RHOBTB2*, is a known tumor suppressor and member of the Rho GTPases subfamily of signaling proteins, consisting of RHOBTB1, RHOBTB2, and RHOBTB3 (5, 6). Rho GTPases act as molecular switches and can revert back and forth between an active, GTP-bound, and an inactive, GDP-bound state (7). Rho proteins become activated through interactions with guanine exchange factors (GEFs) that catalyze the release of bound GDP, subsequently leading to the binding of GTP (7). Termination of Rho signaling is achieved through the hydrolysis of GTP by GTPase-activating proteins (GAPs) (Figure 1) (7). When activated, Rho proteins interact with downstream effectors regulating a variety of cellular processes, including apoptosis, cellular growth, cytoskeletal organization, vesicular transport, and transcription (8, 9). There is evidence suggesting that RHOBTB proteins are tissue-specific, with RHOBTB2 primarily being expressed in neural tissues. However, it is also expressed to a lesser extent in fetal tissues, such as the lungs, heart, and brain (10, 11). Pathological variants in Rho family GTPases, such as RAC1, RAC3, and CDC42, as well as GEFs and GAPs, which affect key developmental processes, are also implicated in a variety of NDDs (12–16).

**Figure 2:**
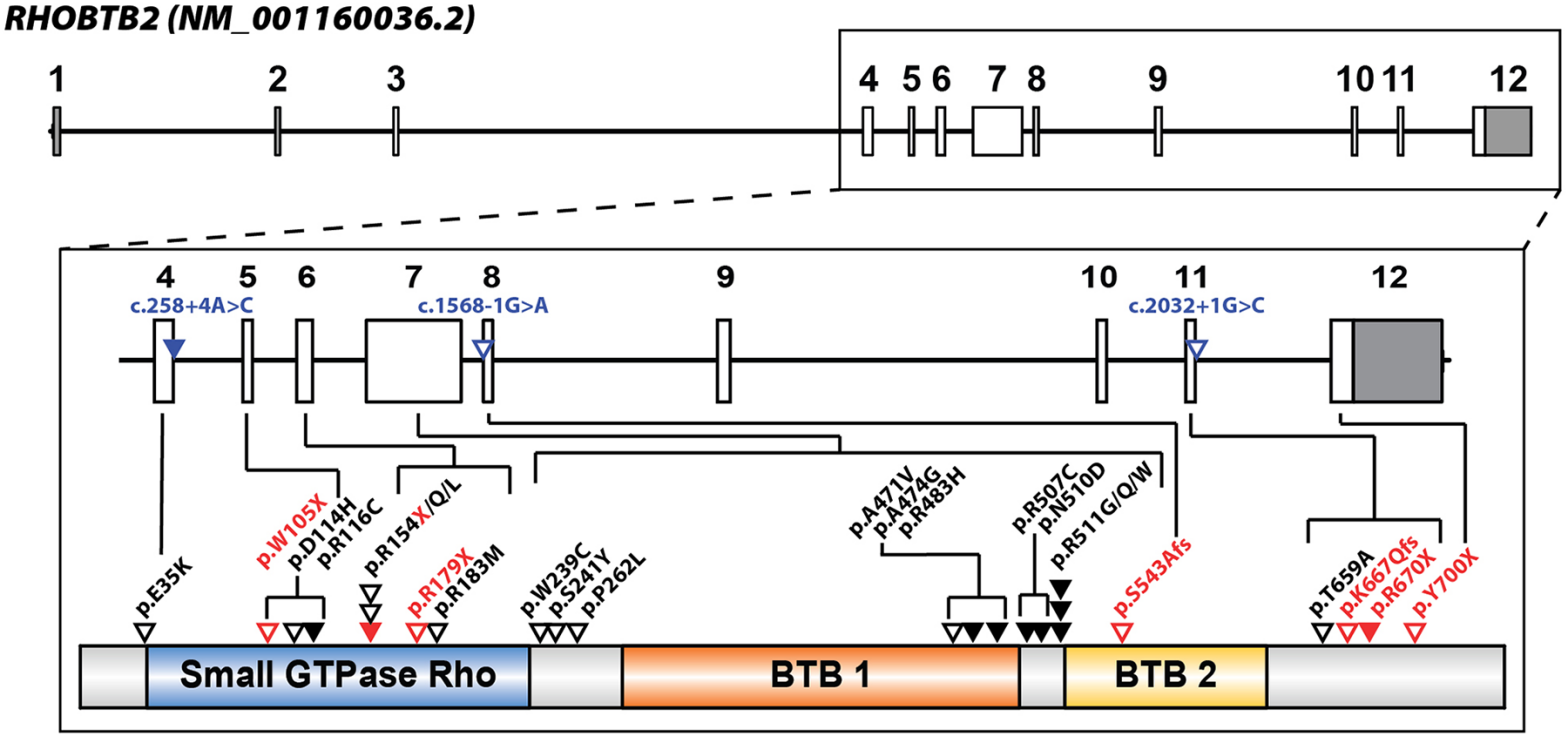
*RHOBTB2* gene and protein structure with locations of patient variants associated with neurodevelopmental disorder (NDD) depicted. RHOBTB2 consists of 12 exons (NM_001160036.2, ENST00000519685.5). The non-coding exons are colored gray, and the coding exons are white (boxes). Multiple patient variants are associated with *RHOBTB2*-related NDD and located in intronic and exonic regions between exon 4 and 12. Intronic or splice variants are labeled in blue and triangles indicate approximate variant locations in relation to exons. Solid triangles indicate multiple individuals reported in the literature with that variant. Missense variants (black) and truncating variants (red) are labeled with triangles in proximity to protein domains (NP_001153508.1). Stacked triangles indicate multiple variants at a specific amino acid residue and slashes delineate all reported amino acid substitutions. The RHOBTB2 protein consists of a Rho-like GTPase region, a BTB1 domain, and a BTB2 domain, based on Pfam (IPR000210) and PANTHER (IPR003578). Variant information was obtained from published reports in the literature (19–27).

RHOBTB2 is considered an atypical Rho protein due to its modular, large size, and overall composition (8, 17, 18). While most Rho proteins exhibit only one Broad-complex, Tramtrack, and Bric-à-brac (BTB) domain, RHOBTB2 has two BTB domains (11). The structure of RHOBTB2 consists of a Rho-like GTPase region at the N-terminus and two BTB domains (Figure 2) (10).

There is some speculation that a proline-rich region after the Rho-like GTPase domain might be a Src homology 3 (SH3) domain-binding site, as SH3 domains are found in proteins involved in signal transduction; however, this has yet to be verified experimentally (28, 29). This could indicate the potential of RHOBTB2 to regulate proteins with SH3 domains if confirmed. Another site of protein-protein interactions is the BTB domains (18). Most notably, BTB domains are involved in post-translational modifications, such as ubiquitination and degradation (29). The two BTB domains present in RHOBTB2 enable the formation of both homodimers and heterodimers (29). Generally, most proteins that dimerize are involved in cellular processes such as enzyme activation, transcriptional cofactor recruitment, and signal transduction; therefore, any disruption in dimer formation could have detrimental effects (19, 30). Lastly, the GTPase and C-terminal regions, along with the second BTB domain, serve as substrate recognition sites (28).

RHOBTB2 is responsible for the recruitment of proteins associated with tumor growth for ubiquitination and subsequent degradation by the 26S proteasome (28). It does so through its association with the cullin 3-dependent ubiquitin ligase complex (Cul3) via its first BTB domain. First, RHOBTB2 binds to specific substrates, facilitating their recognition and interaction with the Cul3-Rbx1 complex (31). Then, the ring-box 1 protein (Rbx1) interacts with ubiquitin-conjugating enzymes (E2) to promote the transfer of ubiquitin from the E2 enzyme to the substrate protein bound to Cul3 and RHOBTB2. This process repeats, resulting in a polyubiquitin chain (18). The resulting polyubiquitin chain marks the substrate, as well as RHOBTB2 itself, for degradation in the 26S proteasome. Dysregulation of the Cul3-RHOBTB2 interaction results in RHOBTB2 instability and inactivation, suggesting that Cul3 serves as a regulatory mechanism for controlling the levels of RHOBTB2 in the cell (29, 32).

## 3 Role of RHOBTB2 in cancer

**Figure 3:**
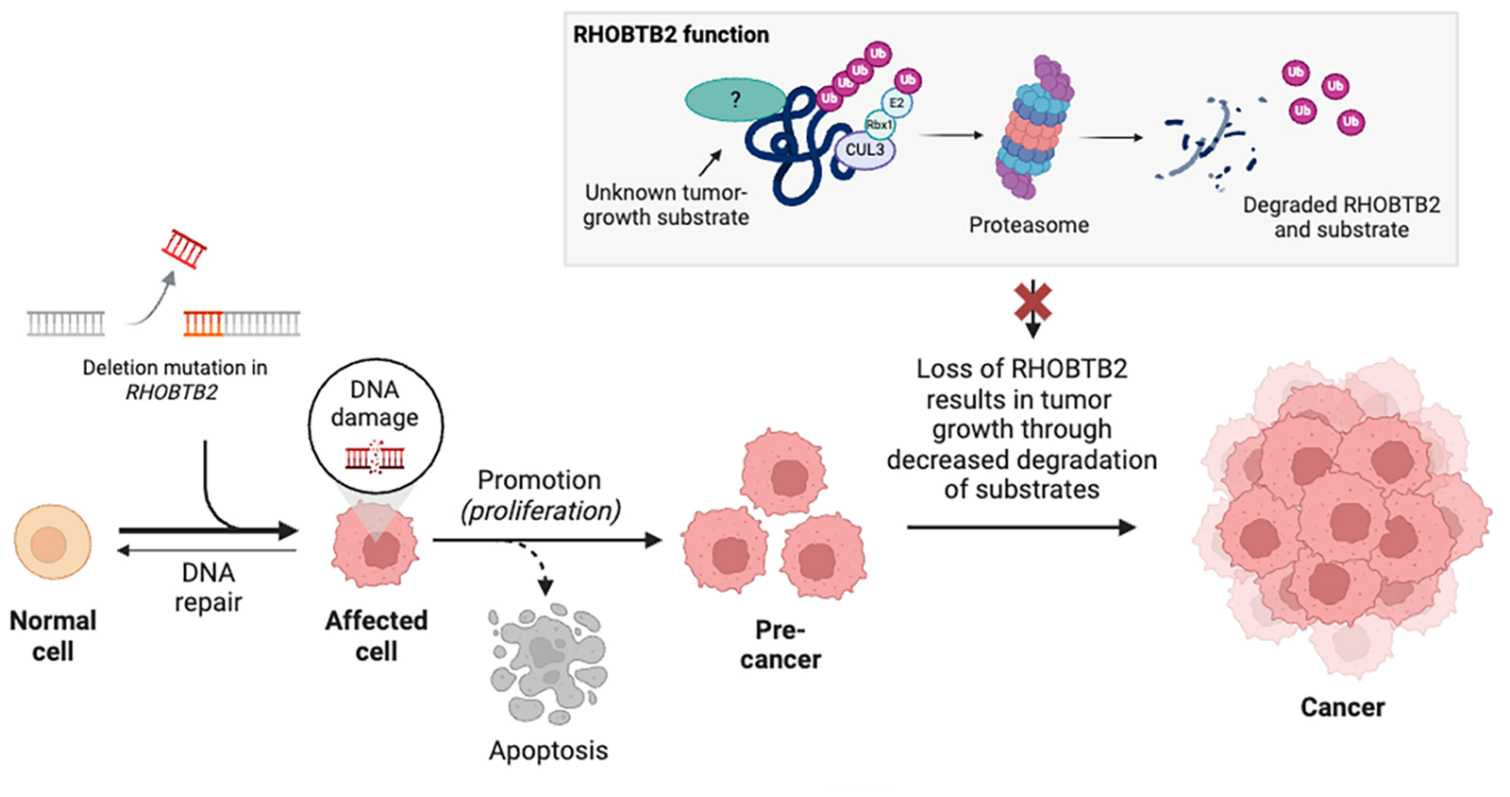
Lack of RHOBTB2-assisted degradation of tumor-growth substrates could lead to increased tumor growth in the absence of RHOBTB2. The exact mechanism as to how deletions and decreased expression of *RHOBTB2* lead to carcinogenesis is unclear, however, one hypothesis is that RHOBTB2 acts as a tumor suppressor by recruiting target proteins, such as CCND1, associated with tumor growth for ubiquitylation and degradation (28, 38, 39). Under normal expression levels, wild-type RHOBTB2 protein acts as a tumor suppressor by recruiting ubiquitination proteins and degradative substrates to a Cul3 ubiquitin ligase complex for degradation (gray box) (28). Deletion variants that lead to a loss of function and/or decreased expression of *RHOBTB2* lead to a lack of sequestration and degradation of cancer-related substrates and therefore unchecked cell cycle growth and cancer (33). Created with BioRender.com.

The RHOBTB family of proteins are tumor suppressors, with diminished or absent expression observed across various cancer types. Specifically, decreased levels of RHOBTB2 have been associated with breast, lung, bladder, and stomach cancers as well as osteosarcomas (5, 33–37). Reduced expression of RHOBTB2 can be caused by epigenetic modification or alternative silencing mechanisms such as the hypermethylation of CpG islands in the promoter region (35). Moreover, deletions and loss-of-function variants in *RHOBTB2* were detected in nearly 10% of breast cancer samples, suggesting that these variants may lead to the loss of tumor-suppressor activity and the initiation of cancerous tumor formation (Figure 3) (33).

The exact mechanism by which RHOBTB2 functions as a tumor suppressor is unclear. One study examining the impact of missense variants on protein function reported that one missense variant associated with cancer (p.Tyr306Asn) resulted in decreased binding to Cul3 and subsequent impaired proteasomal degradation of RHOBTB2 (20). Impaired degradation of RHOBTB2 was not reported in other cancer cell line studies and could indicate alternative cell or tissue-specific mechanisms. Another hypothesized mechanism involves Cyclin D1 (CCND1). CCND1 is another RHOBTB2 interactor that binds within the first BTB domain (38). CCND1 functions in regulating cell cycle progression and transcription of genes involved in cell proliferation (39). It is considered an oncogenic protein as its hyperactivation results in uncontrolled cell proliferation (40). While RHOBTB2 has been observed to arrest growth in breast cancer cells, potentially by downregulating CCND1, some tumor cells proliferate even in the presence of RHOBTB2 (33, 38, 41). One possible explanation is the activation of alternative pathways, such as CCND1-independent mechanisms, that bypass the tumor-suppressing function of RHOBTB2 (33).

## 4 *RHOBTB2*-related neurodevelopmental disorders

Neurodevelopmental disorders (NDD) encompass a range of conditions often characterized by seizures, intellectual disability, abnormal brain development, movement disorders, and other neurological abnormalities (42). In 2013, NDDs were introduced as an all-encompassing disorder category in *The Diagnostic and Statistical Manual of Mental Illnesses* (DSM-5) (43). While epilepsy itself is not classified as an NDD, it is a common comorbidity of NDD that is often severely debilitating and can impact intellectual development and lead to developmental regression (44, 45). *RHOBTB2*-related disorders were first characterized as a severe subtype type of NDD, known as developmental and epileptic encephalopathy (DEE) (19). DEE is characterized by frequent seizures that result in debilitating intellectual and behavioral impairments (46). Before 2018, the role of RHOBTB2 in neurodevelopment had not been studied in detail, however since then, small case studies with one (21), two (22–25), or three patients (26), as well as larger case studies with ten or more patients (19, 20, 27) have been published on NDD related to variants in *RHOBTB2*. Twenty-eight different variants have been identified to cause *RHOBTB2*-related NDD (Figure 2) (19–27). Recurrent variants associated with *RHOBTB2*-related DEE are predominantly *de novo* heterozygous missense variants found within the BTB domain region, such as p.(Arg483His), p.(Arg507Cys), p.(Arg511Gly), p. (Arg511Trp), and p.(Arg511Gln) (19, 20). Notably, three heterozygous variants linked to DEE, p.(Ala471Val), p.(Ala474Gly), and p.(Arg483His), are located in the BTB1 domain, and one homozygous variant, p.(Ser543Alafs*52), is located within the BTB2 domain; the remaining five heterozygous variants are clustered between the two BTB domains (20).

### 4.1 Clinical characteristics

Genotype-phenotype correlations in *RHOBTB2*-related NDDs are exemplified by shared clinical features linked to specific variant clusters, such as early seizure onset in individuals with missense RHOBTB2 variants clustering in the BTB domain region. Notably, two children with the p.(Arg483His) variant developed seizures as early as 4 days after birth (19, 23). Early seizure onset is associated with severe intellectual disability, developmental delay, and motor function issues. Various types of seizures have been reported in different individuals, including febrile seizures, bilateral tonic-clonic, generalized tonic-clonic, and status epilepticus. Another condition commonly reported is microcephaly, which has been linked to seizures, aphasia, and developmental delay (47). Developmental regression has occurred in some cases, including a child with a p.(Arg511Gln) variant, who did not develop symptoms of encephalopathy until the age of 14 (23). Cognitive impairment appears milder in those with a later onset of epilepsy, but significant intellectual regression or stagnation is still present (19, 21, 23, 27). Motor function in these individuals is severely limited and delayed compared to healthy individuals, as assessed by the Centers for Disease Control and Prevention (CDC) developmental milestones (48). Many individuals saw a decrease in motor abilities following the onset of status epilepticus. Motor impairments range from total lack of head control and inability to walk to walking with a broad-based or unsteady gait. Some children develop walking abilities after some time, ranging from as early as 1 year or as late as 7 years old (19, 27). An additional study expanded the phenotypic spectrum for *RHOBTB2*-related disorders to include paroxysmal symptoms similar to those in alternating hemiplegia of childhood (AHC) (27). The study found that 84% of cases were reported to have paroxysmal movement disorders, which were characterized by sporadic involuntary movements, with hemiplegia being one of the most common symptoms (27, 49, 50).

Expanding the genotype-phenotype correlations, individuals with *de novo* heterozygous missense variants in the GTPase domain exhibit a broader spectrum of neurodevelopmental outcomes distinct from those associated with BTB domain variants, ranging from mild to moderate intellectual disability, learning difficulties, to developmental regression (20). Although seizures have been reported in a few of these individuals, antiseizure medications were effective in obtaining seizure control (20). More thorough clinical summaries of these and other patients can be found in the Supplementary Table 1 of Langhammer et al. (2023). The genotype-phenotype correlations in *RHOBTB2*-related NDDs highlight the complexity of these conditions, as variants within different protein domains contribute to a spectrum of neurological manifestations. This emphasizes the need for further research elucidating the underlying mechanisms behind these variants to better inform therapeutic interventions.

### 4.2 Preclinical studies investigating the impact of *RHOBTB2-*related NDD variants

**Figure 4:**
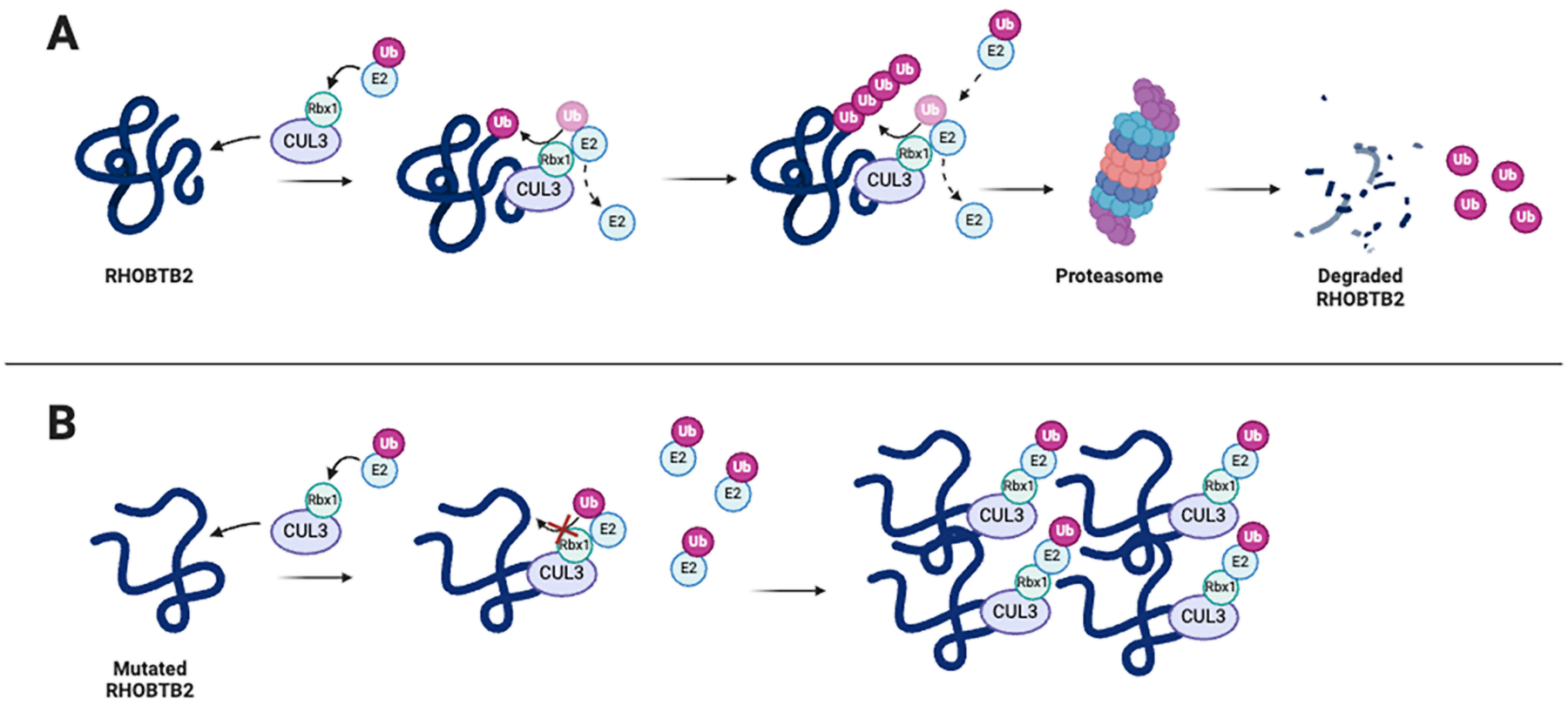
RHOBTB2 containing missense variants in the BTB region leads to improper ubiquitination by Cul3 and increased levels of RHOBTB2 in neural tissues, causing impaired neurodevelopment. **(A)** RHOBTB2 binds to Cul3, a ubiquitin ligase scaffolding protein (31). Cul3 forms a complex with E2 and Rbx1. E2 carries the ubiquitin while Rbx1 transfers the ubiquitin to RHOBTB2 (18). The polyubiquitin chain marks RHOBTB2 for degradation by the 26S proteasome. **(B)** Evidence supports the hypothesis that missense variants in the BTB domains of RHOBTB2 destabilize the protein, thus impairing proper ubiquitination by CUL3 and degradation of RHOBTB2 (19). However, the exact mechanism of action remains unclear. Created with BioRender.com.

The molecular mechanisms associated with RHOBTB2 variants have been generally underexplored, leaving a significant gap in our understanding of their impact on patients. A recent study examined *in silico* structural consequences, as well as Cul3 binding interference and proteasomal degradation of RHOBTB2 using HEK293 cells, to investigate the impact of variants located in the BTB vs. the GTPase domain (20). Their analyses confirmed previous findings that BTB domain variants do not impair Cul3 binding and result in impaired proteasomal degradation of RHOBTB2 (19, 20). For variants located within the GTPase domain, specifically p.(Asp114His), p.(Arg116Cys), p.(Arg154Gln), and p.(Arg154Leu), structural consequences predicted by *in silico* methods suggest that these variants disrupt the electrostatic complementarity of potential protein-protein interactions (20). Additionally, *in vitro* methods revealed these GTPase variants, like the BTB variants, do not impair Cul3 binding (20). However, unlike the BTB variants, GTPase variants did not result in increased RHOBTB2 levels, suggesting an alternative pathogenic effect on RHOBTB2 function (20). In a separate study, mice neuroblastoma cells (Neuro-2a cells) were used to investigate the impaired degradation of mutant RHOBTB2 (26). The variants, p.(Arg483His), p.(Arg507Cys), and p.(Arg511Gln), were transfected into the Neuro-2a cells, and the cells were treated with the proteasome inhibitor MG132. The three RHOBTB2 mutants were found to be more abundant than wild-type RHOBTB2, although the difference was only significant for the p.(Arg507Cys) variant (26). Additionally, Cul3 was coexpressed in Neuro-2a cells to test whether mutant RHOBTB2 protein degradation would increase. The level of the wild-type RHOBTB2 protein decreased with the coexpression of Cul3, but there was little or no change in the level of the variant RHOBTB2 protein (26). These results reinforce the previous findings that RHOBTB2 with variants in the BTB domain can bind to Cul3 but is inadequately degraded by proteasomes. These studies support the hypothesis that increased levels of RHOBTB2 are likely caused by an unstable protein structure resulting from missense variants in the BTB region that affect ubiquitination and degradation, rather than affecting the binding of Cul3 (Figure 4) (19, 26).

To date, no patient-variant animal models have been generated to investigate the neurodevelopmental effects in *RHOBTB2*-related disorders. However, other animal studies support the role of RHOBTB2 in neuronal processes (Table 1) (51). *Drosophila sp. RhoBTB* knockout model flies lacking RhoBTB exhibited seizures and motor degression (19). Conversely, flies overexpressing RhoBTB in neurons exhibited more spasms, hyperactivity, and paralysis compared to control flies, supporting the involvement of RHOBTB2 in neuronal function (19). In a study involving prenatal malnourished rats, *RHOBTB2* was identified as a gene that modulates neurodevelopment (51). The primary objective of the study was to investigate the impact of maternal prenatal malnutrition on neurodevelopment and identify genes involved in regulating neurodevelopment. The researchers analyzed the growth of rat hippocampus and prefrontal cortex tissue to determine deficiencies. Using a weighted gene co-expression network analysis, this research confirmed the role of RHOBTB2 in synaptic development, neuronal projection, and cognitive functions (51). The positive correlation between RHOBTB2 expression and the development of hippocampal and prefrontal tissue supported its role in these processes. Additionally, the study found a potential regulatory link between *RHOBTB2* and the transcription factor gene *CEBPA* (CCAAT/enhancer-binding protein-alpha), which is involved in cell proliferation and termination (51). The CEBPA protein may promote the transcription of *RHOBTB2*, however, further studies need to be done to validate and fully understand the potential of upregulating *RHOBTB2* through CEBPA modulation (51, 53).

**Table 1:** Models of *RHOBTB2*-related NDDs and DEE.

Model	Method	Results	Publication
Fruit Fly (*Drosophila melanogaster*) - single ortholog (*RhoBTB*) of vertebrate paralogs	Overexpression of *RhoBTB*	Flies exhibited hyperactivity and paralysis	(19)
	Knockdown of *RhoBTB*	No or milder hyperactivity and paralysis	
HEK293 cells	His-Myc-tagged wild-type and mutant *RHOBTB2* treated with proteasome inhibitor	Increased levels of RHOBTB2 with BTB variants compared to wild-type due to impaired degradation; no detected impairment to Cul3 binding	(19)
Neuro-2a cells	Treated with a proteasome inhibitor or DMSO as a control	Confirmed Straub et al. (2018) findings that mutant RHOBTB2 with BTB variants escaped degradation mediated by Cul3 complex	(26)
Rats, Sprague-Dawley	Co-expression network analysis identifying genes affected by prenatal nutrition deficiency	Confirmed role in synaptic development and cognitive function; identified *Cebpa* as a transcriptional regulatory factor of RHOBTB2	(51)
HEK293 cells	His-Myc-tagged wild-type and mutant *RHOBTB2* treated with proteasome inhibitor	Confirmed Straub et al. (2018) findings regarding BTB variants; GTPase variants do not impair binding to Cul3 nor do they impact RHOBTB2 levels compared to wild-type	(20)

*Drosophila* models revealed hyperactivity and seizure susceptibility associated with overexpression of *RhoBTB* (19). HEK293 cell models were used to investigate the impact of BTB variants on the function of RHOBTB2, the results of which were also confirmed in Neuro-2a cells, which have high *RHOBTB2* expression (11, 19, 52). A rat model, used for exploring prenatal malnutrition's neurodevelopmental risks, further validated the role of RHOBTB2 in learning and synaptic development and uncovered *Cebpa*'s regulatory role in *RHOBTB2* expression (51). HEK293 cells were used again in 2023 to investigate how the location of the variant impacts protein function, including variants outside the BTB region (20).

### 4.3 RHOBTB2 and its role in epileptic encephalopathy

*In vitro* data suggest that certain RHOBTB2 variants contribute to impaired RHOBTB2 degradation, leading to its accumulation, while animal model data supports the role of RHOBTB2 in proper neuronal development and function (19, 51). However, it remains unknown how increased levels of RHOBTB2 in neurons lead to developmental epileptic encephalopathy (DEE). Epilepsy is the result of abnormal neuronal activity in the brain, and the apoptosis pathway is one of many mechanisms that can contribute to the development of epilepsy and seizure susceptibility (54). RHOBTB2 is implicated in apoptosis as it serves as a molecular target for eukaryotic transcription factor 1 (E2F1) (55). E2F1 has a unique role among the E2F transcription factors in inducing pro-apoptotic genes and repressing pro-apoptotic genes. Moreover, there is a direct correlation between levels of E2F1 and RHOBTB2; overexpression of E2F1 leads to increased RHOBTB2, whereas reducing E2F1 leads to decreased RHOBTB2 levels (55). Given this relationship between E2F1 and RHOBTB2, it is not surprising that cells overexpressing RHOBTB2 exhibit higher levels of pro-apoptotic proteins, such as Bax and Bak (56). Notably, alterations in the expression of pro-apoptotic genes have been reported in hippocampal and neocortical tissue taken from patients with pharmacoresistant epilepsy, although apoptotic cells are rarely found, suggesting concurrent anti-apoptotic signaling changes (54). Therefore, overexpression of RHOBTB2 could result in altered cellular apoptosis, presenting a potential pathological mechanism for *RHOBTB2*-related disorders (26). Additional studies are needed to test this hypothesis and gain insights into underlying DEE-related mechanisms.

## 5 Treatment approaches

Modulation of RHOBTB2 levels could potentially be a promising treatment for certain cancers and *RHOBTB2*-related disorders. For example, ectopic expression of RHOBTB2 was shown to inhibit migration and metastasis in breast cancer cell lines (57). However, no RHOBTB2-targeted therapies have been developed to treat cancer.

The current treatment approaches for *RHOBTB2*-related DEE primarily involve the administration of antiseizure medications, with the most frequently prescribed drugs including valproic acid, levetiracetam, topiramate, and oxcarbazepine to manage seizures (19, 21–23, 26, 27). There remains a lack of effective medications targeting symptoms related to motor function and movement disorders. *In vitro* functional studies suggest that BTB domain variants linked to *RHOBTB2*-related disorders lead to RHOBTB2 accumulation, suggesting a potential therapeutic avenue could involve the downregulation of RHOBTB2 expression (19, 20).

The use of artificial intelligence (AI) to investigate molecular-level therapies shows promise in identifying drug candidates capable of binding to and modulating proteins involved in RHOBTB2 signaling. AI systems offer a computational approach to identifying novel gene-related therapies, especially those suitable for drug repurposing (58). For instance, the AI program *mediKanren* was used to identify drug modulators of RHOBTB2, which could serve as treatments for patients with gain-of-function variants (59). By focusing on E2F1's ability to downregulate *RHOBTB2*, the researchers identified drugs that downregulate E2F1, thereby indirectly inhibiting RHOBTB2 expression. One of these drugs was Celecoxib (Celebrex), an FDA-approved nonsteroidal anti-inflammatory drug (NSAID) capable of crossing the blood-brain barrier (60). Celecoxib, commonly used to treat symptoms of arthritis and pain, was found to downregulate *E2F1* and cyclin D1 (60, 61). This downregulation of *E2F1* could potentially reduce the overexpression of RHOBTB2, while the downregulation of cyclin D1 could enhance the tumor suppression function of *RHOBTB2* (55, 61). Other NSAIDs, such as diclofenac and indomethacin, have also been linked to the downregulation of E2F1 target genes, and therefore may also alleviate the effects of gain-of-function variants in RHOBTB2 (55, 61, 62). Furthermore, the use of NSAIDs as a therapeutic approach avoids compromising the tumor suppressor function of RHOBTB2, as evidenced by studies in mice demonstrating the antiproliferative effects of NSAIDs in ovarian cancer (55, 61, 62). However, these studies were tissue-specific and additional preclinical studies are needed to assess their applicability to different cancer types.

Gene therapies that specifically target the variant allele in *RHOBTB2*-related disorders offer future promise and unique challenges. A recent study that is still undergoing development discussed the development of a new antisense oligonucleotide (ASO) therapy that targets RHOBTB2, with the goal of reversing the accumulation of RHOBTB2 to benefit patients with DEE (63). ASOs are short, synthetic strands of nucleic acids designed to bind to specific mRNA sequences, potentially modulating gene expression. However, the rarity of these disorders and the complexity of RHOBTB2's involvement in neural development complicate the development of targeted therapies.

## 6 Discussion

Precise RHOBTB2 expression levels are key to preventing tumor growth and promoting proper nervous system development and function, however, the role of RHOBTB2 in these processes requires further investigation. Studies examining RHOBTB2 as a tumor suppressor show that decreased levels of RHOBTB2, through variants, epigenetic modifications, or silencing mechanisms, are associated with multiple cancers (28, 29, 31, 33, 36). However, the extent of tumor growth induced by RHOBTB2 downregulation and whether RHOBTB2 acts in a tissue-specific manner remains unknown (33). Furthermore, although a few proteins associated with tumor growth have been identified as substrates of the RHOBTB2-Cul3/Rbx1 complex, the exact mechanisms by which RHOBTB2 functions as a tumor suppressor remain unclear.

Alternatively, variants in RHOBTB2 that lead to elevated RHOBTB2 levels are associated with developmental and epileptic encephalopathies (DEE) (19, 20). However, the exact mechanism by which these variants lead to DEE is understudied. One hypothesis from *in vitro* studies suggests that certain RHOBTB2 variants may destabilize the RHOBTB2 protein, impairing its degradation and interactions with other proteins (19, 26). Overall, there is a lack of preclinical and animal model studies investigating variant impact and therapeutic options for *RHOBTB2*-related disorders (Table 1). Currently, only a few missense variants of RHOBTB2 have been studied in animal models, and further research is needed to understand the impact of other variants on RHOBTB2 ubiquitination and degradation, particularly those located outside the BTB region and in the Rho-like region of the RHOBTB2 protein (Figure 2).

## 7 Conclusion

In both cancer and NDD, RHOBTB2 appears to play a role in the targeted degradation of key proteins related to tumor growth and/or neural development. However, the identification of these proteins and whether other mechanisms are involved is still under investigation. Therefore, further research is warranted to delve into the interactions of RHOBTB2 and degradative pathways.

Research into the pathophysiological disease mechanisms associated with expression levels of RHOBTB2 requires further investigation. While RHOBTB2 is linked to cancer onset in cases of decreased expression and neurodevelopmental disorders in potential gain-of-function and loss-of-function scenarios, it remains relatively understudied, with limited information available on its function and disease mechanisms. There is a consistent association between decreased levels of RHOBTB2 leading to tumor growth in specific tissues, but the mechanisms of tumor suppression are unclear. Increased levels of RHOBTB2 have been linked to DEE, but the role of RHOBTB2 in nervous system development is understudied. Future studies exploring the impact of individual patient variants on RHOBTB2 stability and function will contribute valuable insights to our understanding of its role in health and disease. Lastly, there is a need for further *in vivo* studies to identify new treatment avenues for both cancer and DEE, as well as paroxysmal movement disorders associated with *RHOBTB2*-related disorders.
